# The availability of drug by liposomal drug delivery: Individual kinetics and tissue distribution of encapsulated and released drug in mice after administration of PEGylated liposomal prednisolone phosphate

**DOI:** 10.1007/s10637-018-0708-4

**Published:** 2018-12-13

**Authors:** Evelien A. W. Smits, José A. Soetekouw, Ebel H. E. Pieters, Coen J. P. Smits, Nicolette de Wijs-Rot, Herman Vromans

**Affiliations:** 1 Department of Pharmaceutical Sciences, Utrecht University, P.O. Box 80082, 3508 TB Utrecht, The Netherlands; 2 Quality Control, Nutricia Early Life Nutrition, Cuijk, The Netherlands; 3 Holland Innovative, Eindhoven, The Netherlands; 4 Education and Student Affairs, Eindhoven University of Technology, Eindhoven, The Netherlands; 5 Department of Clinical Pharmacy, Division of Laboratory Medicine & Pharmacy, University Medical Centre Utrecht, Utrecht, The Netherlands

**Keywords:** Drug availability, In vivo drug release, Liposomal prednisolone phosphate, Tissue influx, Pharmacokinetics, Targeted drug delivery

## Abstract

Lately, the usefulness of liposomal drug delivery systems has been debated. To better understand the underlying pharmacokinetics of the targeted drug delivery by liposomes, individual encapsulated and non-encapsulated drug concentrations in blood, tumor, liver, spleen and kidneys were quantified after i.v. administration of liposomal prednisolone phosphate in mice. Kinetic analysis shows that the tumor influx of encapsulated drug is not dominant compared to the uptake by the other tissues. Further, from a quantitative point of view, the availability of non-encapsulated drug in the tumor tissue after liposomal delivery is not pronounced as compared to the other tissues studied. However, drug release in the tumor seems more extended than in the other tissues and the non-encapsulated drug concentration decreases more slowly in the tumor than in the liver and spleen. The spleen shows a high affinity for the uptake of encapsulated drug as well as the release of drug from the liposomes. Subsequently, released drug in the spleen, and possibly also in other tissues, is probably quickly redistributed towards the blood and other tissues. This also impairs the drug delivery effect of the liposomes. In contrast to the released drug in the central circulation, liver and spleen, the released drug concentration in the tumor remains at a fairly constant level likely due to the extended release kinetics from the liposomes. These extended release characteristics in the tumor most probably contribute to the beneficial effect. Nevertheless, it should be noted that larger released drug concentrations are formed in healthy tissues.

**Electronic supplementary material:**

The online version of this article (10.1007/s10637-018-0708-4) contains supplementary material, which is available to authorized users.

## Introduction

Tumor targeting by liposomes has been considered a promise for quite a few decades now and can increase the therapeutic index [1, 2]. Drug targeting to tumors by liposomes has been assumed to depend on the enhanced permeation and retention (EPR) effect [2–4]: due to their specific size the liposomes should not extravasate into healthy tissues and should avoid renal clearance, whereas wide fenestrations in the leaky tumor vasculature would allow the liposomes to permeate into the tumor tissue. In addition, the absence of well-functioning lymphatic drainage in the tumor should result in enhanced tumor retention. In this respect, a stealth coat of hydrophilic polymers like polyethylene glycol (PEG) is important to delay the uptake by the phagocytes of the mononuclear phagocyte system (MPS) and to attain a blood circulation time long enough for the nanoparticles to reach the tumor tissue.

Lately, however, the success of tumor targeted delivery by nanomedicines including liposomes and the corresponding EPR effect has been questioned [4–6]. One of the issues is that PEGylated liposomes still localize considerably in healthy tissues like the liver and the spleen [7–9]. It is also pointed out that efficacy and toxicity can only be related to the released, non-encapsulated drug and not to the drug that is still encapsulated in the liposomes. Hence, the availability of the released drug and the corresponding fate (i.e. retention, distribution, elimination) are as least as important as the behavior of the liposomal carrier.

To understand and improve the pharmacokinetics of targeted drug delivery by liposomes, the separate quantitation of the drug that is still encapsulated in the liposomes (further referred to as encapsulated drug) and the released drug is essential, since efficacy and toxicity can only be related to the level of the released drug as discussed above. While techniques, which use the different physicochemical properties of the liposome and the drug like charge, size and hydrophobicity, were useful for the separate quantification of encapsulated and released drug in plasma [10–16], these techniques are not suitable for the separate quantification in tissues. Homogenization is required prior to their application, which induces liposome rupture, release of encapsulated drug and, consequently, overestimations of the released drug concentration [17].

Laginha et al. defined a creative approach to approximate bioavailable, released doxorubicin levels in tumor tissue after intravenous administration of Doxil: although the accuracy is uncertain, the cell nucleus acts like a sink for released doxorubicin and was used as a measure for the bioavailable drug concentration [18]. Alternatively, microdialysis can be applied to measure the non-protein bound drug in tissue fluids only through passive diffusion from the interstitial fluid across the semi-permeable membrane of the microdialysis catheter [19]. Also the measurement of the lipid/drug ratio can provide some insights in the in vivo release [20]. Unfortunately, the drug/lipid ratio cannot differentiate between encapsulated drug and released drug that is still present in the tissue [21], but the released drug in the tissue is what should be known as discussed above. Without devaluing the aforementioned methods, the encapsulation of a phosphate prodrug like prednisolone phosphate (PP) into liposomes does enable the direct and accurate quantification of encapsulated and released drug in tissues [22–24] as follows. The differentiation of encapsulated and released drug is attained by the rapid dephosphorylation of PP in vivo [22, 25, 26]. The conversion of PP into prednisolone (P) after release from the liposome in whole blood [27] and various tissues, i.e. liver and kidneys [23], is determined to be instantaneously. Phosphatases are also overexpressed in tumor microenvironments [28]. Moreover, it is strongly believed that after liposome uptake by macrophages, the encapsulated drug is liberated in the endosomal/lysosomal compartment and hydrolyzed into P [29–31], because phosphatases are also present in macrophages and lysosomes [32]. Thus, it is assumed that PP is rapidly converted into P after release. Consequently, the in vivo PP concentration represents the encapsulated drug concentration and the in vivo P concentration represents the released drug concentration [22, 23, 27]. N.b. PEGylated liposomes containing PP showed to reduce the tumor growth in mice in contradiction to the free drug formulation [9].

To our knowledge, accurate released drug concentrations in solid tumors have rarely been compared to such concentrations in healthy tissues until now. From an efficacy/toxicity point of view, this is very essential as discussed above. Therefore, in this study, PEGylated liposomal PP was used as a model formulation in mice to quantify the encapsulated and released drug concentrations in the tumor tissue as well as in whole blood, liver, spleen and kidneys, for which previously large liposome concentrations were observed [9, 33]. To further understand the pharmacokinetics, the in vivo tissue influx of encapsulated drug and the in vivo drug release from the liposomes are calculated for each of the tissues separately using kinetic modelling. The results provide quantitative data of the pharmacokinetics of liposomal targeted drug delivery and demonstrate the quantitative availability of the released drug in different tissues.

## Materials and methods

### Materials

All materials were used as received. Dipalmitoyl phosphatidyl choline (DPPC) and PEG2000-distearoyl phosphatidyl ethanolamine (PEG2000-DSPE) were purchased from Lipoid GmbH (Ludwigshafen, Germany). Alkaline phosphatase from rabbit intestine, cholesterol, dexamethasone (D), dexamethasone disodium phosphate and prednisolone were purchased from Sigma (St. Louis, MO, USA). Prednisolone disodium phosphate was purchased from Bufa (IJsselstein, The Netherlands). Methanol HPLC gradient grade, which was used during sample preparation, was purchased from Mallinckrodt Baker BV (Deventer, The Netherlands).

### Liposome preparation and characterization

PEGylated liposomes encapsulating prednisolone phosphate were prepared using the film-extrusion method as described by Metselaar et al. starting from a mixture of DPPC, cholesterol and PEG2000-DSPE in a molar ratio of 1.85:1.0:0.15, respectively [22].

During liposome characterization total, encapsulated and non-encapsulated PP concentrations in the liposome preparation were determined using a previously developed method [34], in which the non-encapsulated PP was distinguished from the encapsulated PP by dephosphorylation into P using alkaline phosphatase. Mean liposome sizes were determined by dynamic light scattering as also described previously [34].

### Murine tumor model

Male C57Bl/6J (15 mice; 20–25 g) were obtained from Charles River (The Netherlands). The mice were kept in standard housing with standard rodent chow and water available ad libitum on a 12 h light/dark cycle. Experiments were performed according to all applicable international, national, and/or institutional guidelines and were approved by the animal experiment committee of Utrecht University. For tumor induction, 1 × 10^6^ murine B16F10 melanoma cells were inoculated subcutaneously in the flank of the mice. Tumor size was monitored manually and the tumor volume (V_T_) was calculated by applying Eq. (1):

1 $$ {\mathrm{V}}_{\mathrm{T}}=0.5\times {\mathrm{a}}^2\times \mathrm{b} $$

where a is the smallest diameter and b is the largest diameter.

### Medication and sampling

At a tumor volume of 3 × 10^2^ mm^3^ ± 1.5 × 10^2^, 14 mice received 36 μmol/kg PP (=18 mg/kg of prednisolone disodium phosphate) by tail vein injection of liposomal PP. One mouse was not administered with liposomal PP and served as control. At distinct time intervals after injection blood was sampled (~200 μL) via cheek puncture into EDTA containing tubes. Subsequently, the specific animal was sacrificed by cervical dislocation and tumor, liver, spleen and kidneys were dissected. The tissues were weighted and all samples were stored at −20°C to prevent significant dephosphorylation of PP after sampling.

### Analytical methodology

The collected blood and tissue samples were processed and analyzed according to previously developed and validated methodology for the quantitative differentiation of encapsulated PP and released P in murine whole blood and liver tissue [27, 35]. The suitability of this methodology for tumor, kidney and splenic tissue was verified by qualification (internal study similar to the validation as described by Smits et al. [35]). As discussed in the introduction, the encapsulation of a phosphate prodrug like PP into liposomes enables the direct and accurate quantification of encapsulated and released drug in tissues: as long as the compound is encapsulated it is assessed as a phosphate. When released in the tissue, conversion to the parent steroid is so quick that the level of this compound can be regarded as the amount of released drug.

To summarize above methodology, blood samples were removed from the freezer and thawed for 30 min only prior to sample preparation, whereas tissue samples were processed while still frozen. In this way significant dephosphorylation of PP after sampling and, consequently, significant overestimations of the released drug concentration are prevented. Hereafter, the sample preparation of whole blood samples involved protein precipitation with four equivalents of methanol containing the internal standards D and dexamethasone phosphate (DP). To do so, 100 μL of the blood samples were used. Tissue samples were homogenized in 10 mL methanol/g tissue and again the methanol contained the internal standards. The use of such amounts of methanol ensures complete liposome rupture and prevents dephosphorylation of PP that is released during sample preparation. After the samples were treated with high intensity focused ultrasound to disrupt the cell membranes, PP and P concentrations were measured by LC-MS analysis. The used chromatographic conditions were as described by Smits et al. [35]. Comprehensive liquid chromatography together with negative electrospray ionization, insource-fragmentation of P and D and high-resolution accurate mass Orbitrap-MS analysis was used to avoid the significant interference by (biological) impurities from the complex matrix during PP and P detection. The selectivity, sensitivity and quantitative accuracy of the methodology is sufficient for the quantification of PP and P in murine blood, liver, tumor, spleen and kidneys. Peak areas of co-eluting impurities in blank samples are <20% for PP and P and < 5% for DP and D compared to the lowest peak areas in calibration standards and quality control (QC) samples. The quantitative accuracy of the methodology within the used range is 80–120%.

The tumor calibration standards served also as QC samples and were prepared by using the tumor from the control mouse. Note, all calibration standards were prepared using non-encapsulated PP instead of liposomal PP in favor of the precision.

### Data analysis and statistics

#### Correction of PP and P tissue concentrations for residual blood

The PP and P tissue concentrations that were measured by LC-MS were corrected for the PP and P in the residual blood. Because a large part of the blood in the dissected tissues is already gone after euthanasia, the literature values according to Brown et al. do not apply [36]. Since the observed large blood concentrations especially with regard to the encapsulated concentrations (see Results section) can yield overestimations, the tissue concentrations were corrected as follows. At five minutes after i.v. administration (0.08 h), PEGylated liposomes are assumed to be homogenously distributed over the circulation but are also assumed still to be located in the circulation only. This is supported by the observation that the distribution volume of prednisolone phosphate in PEGylated liposomes is close to the plasma volume in rats and humans with arthritis [22, 30]. For PEGylated liposomal doxorubicin similar results were found [7].

Using the above assumptions, the volume fraction of residual blood (VFB) was calculated for each tissue of interest by applying Eq. (2):

2 $$ V\mathrm{FB}=\frac{\mathrm{measured}\ \mathrm{C}{(0.08)}_{\mathrm{PPX}}}{\mathrm{C}{(0.08)}_{\mathrm{PPB}}} $$

where “measured C(0.08)_PPX_” is the uncorrected encapsulated PP concentration as measured by LC-MS in the tissue “X” at *t* = 0.08 h, and where C(0.08)_PPB_ is the encapsulated PP blood concentration at *t* = 0.08 h. Then, the measured PP and P tissue concentrations were corrected for the PP and P in the residual blood by applying Eq. (3) for each time t:

3 $$ {\mathrm{C}}_{\mathrm{P}\left(\mathrm{P}\right)\mathrm{X}}=\frac{\mathrm{measured}\ {\mathrm{C}}_{\mathrm{P}\left(\mathrm{P}\right)\mathrm{X}}-{\mathrm{C}}_{\mathrm{P}\left(\mathrm{P}\right)\mathrm{B}}\times \mathrm{VFB}\ }{\left(1-\mathrm{VFB}\right)} $$

where C_P(P)X_ is the corrected PP or P tissue concentration at time t, “measured C_P(P)X_” is the uncorrected PP or P concentration as measured by LC-MS in the tissue “X” at time t, and C_P(P)B_ is the corresponding PP or P blood concentration at time t.

Whole blood and tissue densities are assumed to be 1 g/mL.

#### Outliers

Data derived from two subjects for which an extremely poor total drug recovery was observed were considered to be outliers and were excluded. For these subjects the recovery was a factor 3–22 lower compared to adjacent subjects when plotted versus time. Most likely, the animals were injected incorrectly. Injection in the tail vein is a delicate exercise because of the small size. Except for these two, no outliers were removed.

The tumor tissue dissected at *t* = 16.5 h was lost during sample preparation. Therefore, the corresponding tumor concentration is lacking.

#### Regression of encapsulated PP and released P concentrations

The encapsulated PP and released P blood concentrations were fitted by least squares non-linear regression with a 95% confidence interval using Eq. (4) and (5) (Minitab 17 Statistical Software, Minitab Inc., State College, PA, USA), which describe straightforward first-order kinetics:

4 $$ {\mathrm{C}}_{\mathrm{PPB}}\left(\mathrm{t}\right)={\mathrm{C}}_{\mathrm{PPB}}(0)\times {\mathrm{e}}^{-{\mathrm{k}}_{\mathrm{PPB}}\times \mathrm{t}} $$

5 $$ {\mathrm{C}}_{\mathrm{PB}}\left(\mathrm{t}\right)={\mathrm{C}}_{\mathrm{PB}}(0)\times {\mathrm{e}}^{-{\mathrm{k}}_{\mathrm{PB}}\times \mathrm{t}} $$

where C_PPB_(t) and C_PB_(t) are the encapsulated PP and released P blood concentration at time t, C_PPB_(0) and C_PB_(0) are the pseudo initial encapsulated PP and released P concentrations, and k_PPB_ and k_PB_ are the first-order decline rate constants for encapsulated PP and released P in blood, respectively. Normality of the residuals was verified (*p* > 0.05).

The encapsulated PP concentrations in the tissues of interest were fitted as follows. First, differential equations describing the kinetics of encapsulated PP in tissue “X” were generated. The influx of encapsulated PP from the blood is assumed to be a first order process, whereas the decline of encapsulated PP in the tissue is assumed to be a zero order (Eq. (6)) or first order (Eq. (7)) process:

6 $$ \frac{\mathrm{d}\left[{\mathrm{m}}_{\mathrm{X}}\times {\mathrm{C}}_{\mathrm{PPX}}\left(\mathrm{t}\right)\right]}{\mathrm{d}\mathrm{t}}={\mathrm{k}}_{\mathrm{PPB}\mathrm{X}}\times {\mathrm{V}}_{\mathrm{DPP}}\times {\mathrm{C}}_{\mathrm{PPB}}\left(\mathrm{t}\right)-{\mathrm{R}}_{\mathrm{PPX}} $$

7 $$ \frac{\mathrm{d}\left[{\mathrm{m}}_{\mathrm{X}}\times {\mathrm{C}}_{\mathrm{PPX}}\left(\mathrm{t}\right)\right]}{\mathrm{d}\mathrm{t}}={\mathrm{k}}_{\mathrm{PPB}\mathrm{X}}\times {\mathrm{V}}_{\mathrm{DPP}}\times {\mathrm{C}}_{\mathrm{PPB}}\left(\mathrm{t}\right)-{\mathrm{k}}_{\mathrm{PPX}}\times {\mathrm{m}}_{\mathrm{X}}\times {\mathrm{C}}_{\mathrm{PPX}}\left(\mathrm{t}\right) $$

where C_PPX_ is the encapsulated PP concentration in the tissue, m_x_ is the mass of the tissue corrected for the mass of the residual blood by: m_x_ = “mass uncorrected” x (1-VFB), k_PPBX_ is the first order rate constant corresponding to the influx of encapsulated PP from the blood, V_DPP_ is the volume of distribution of encapsulated PP, R_PPX_ is the zero order decline rate constant and k_PPX_ is the first order decline rate constant. These differential equations were solved over time t yielding the following equations:

8 $$ {\mathrm{C}}_{\mathrm{PPX}}\left(\mathrm{t}\right)=\frac{{\mathrm{k}}_{\mathrm{PPB}\mathrm{X}}}{{\mathrm{k}}_{\mathrm{PPB}}}\times \frac{{\mathrm{V}}_{\mathrm{DPP}}}{{\mathrm{m}}_{\mathrm{X}}}\times {\mathrm{C}}_{\mathrm{PPB}}(0)\times \left(1-{\mathrm{e}}^{-{\mathrm{k}}_{\mathrm{PPB}}\times \mathrm{t}}\right)-\frac{1}{{\mathrm{m}}_{\mathrm{X}}}\times {\mathrm{R}}_{\mathrm{PPX}}\times \mathrm{t} $$

9 $$ {\mathrm{C}}_{\mathrm{PPX}}\left(\mathrm{t}\right)=\frac{{\mathrm{k}}_{\mathrm{PPB}\mathrm{X}}}{{\mathrm{k}}_{\mathrm{PPX}}-{\mathrm{k}}_{\mathrm{PPB}}}\times \frac{{\mathrm{V}}_{\mathrm{DPP}}}{{\mathrm{m}}_{\mathrm{X}}}\times {\mathrm{C}}_{\mathrm{PPB}}\left(\mathrm{t}\right)-\frac{{\mathrm{k}}_{\mathrm{PPB}\mathrm{X}}}{{\mathrm{k}}_{\mathrm{PPX}}-{\mathrm{k}}_{\mathrm{PPB}}}\times \frac{{\mathrm{V}}_{\mathrm{DPP}}}{{\mathrm{m}}_{\mathrm{X}}}\times {\mathrm{C}}_{\mathrm{PPB}}(0)\times {\mathrm{e}}^{-{\mathrm{k}}_{\mathrm{PPX}}\times \mathrm{t}} $$

However, as is discussed in the Supplementary material (see ESM 1), the encapsulated PP tumor kinetics is better described by the following differential equations expressing the change of the encapsulated PP tumor concentration with time:

10 $$ \frac{\mathrm{d}{\mathrm{C}}_{\mathrm{PPT}}\left(\mathrm{t}\right)}{\mathrm{d}\mathrm{t}}={\mathrm{k}}_{\mathrm{PPB}\mathrm{T}}\times {\mathrm{C}}_{\mathrm{PPB}}\left(\mathrm{t}\right)-{\mathrm{R}}_{\mathrm{PPT}} $$

11 $$ \frac{\mathrm{d}{\mathrm{C}}_{\mathrm{PPT}}\left(\mathrm{t}\right)}{\mathrm{d}\mathrm{t}}={\mathrm{k}}_{\mathrm{PPB}\mathrm{T}}\times {\mathrm{C}}_{\mathrm{PPB}}\left(\mathrm{t}\right)-{\mathrm{k}}_{\mathrm{PPT}}\times {\mathrm{C}}_{\mathrm{PPT}}\left(\mathrm{t}\right) $$

These differential equations were solved over time t yielding the following equations:

12 $$ {\mathrm{C}}_{\mathrm{PPT}}\left(\mathrm{t}\right)=\frac{{\mathrm{k}}_{\mathrm{PPB}\mathrm{T}}}{{\mathrm{k}}_{\mathrm{PPB}}}\times {\mathrm{C}}_{\mathrm{PPB}}(0)\times \left(1-{\mathrm{e}}^{-{\mathrm{k}}_{\mathrm{PPB}}\times \mathrm{t}}\right)-{\mathrm{R}}_{\mathrm{PPT}}\times \mathrm{t} $$

13 $$ {\mathrm{C}}_{\mathrm{PPT}}\left(\mathrm{t}\right)=\frac{{\mathrm{k}}_{\mathrm{PPB}\mathrm{T}}}{{\mathrm{k}}_{\mathrm{PPT}}-{\mathrm{k}}_{\mathrm{PPB}}}\times {\mathrm{C}}_{\mathrm{PPB}}\left(\mathrm{t}\right)-\frac{{\mathrm{k}}_{\mathrm{PPB}\mathrm{T}}}{{\mathrm{k}}_{\mathrm{PPT}}-{\mathrm{k}}_{\mathrm{PPB}}}\times {\mathrm{C}}_{\mathrm{PPB}}(0)\times {\mathrm{e}}^{-{\mathrm{k}}_{\mathrm{PPT}}\times \mathrm{t}} $$

Then, the encapsulated PP concentrations in the tissues of interest were fitted using least squares non-linear regression with a 95% confidence interval using Eq. (8) and (9) or (12) and (13). If necessary, statistical weights were included to correct for an unequal distribution of data points. Further, the normality of the residuals was verified (*p* > 0.05) and the curve fit with the smallest S-value (standard error of the regression) was preferred.

Differences between the curve fits of encapsulated PP were accepted to be statistically significant when there was no overlap of the corresponding 95% confidence intervals. Significant differences between the released P concentrations were evaluated using linear regression of the released P concentrations in blood or the specific tissue as a function of the released P concentrations in the (other) tissues.

#### Calculation of tissue influx

The rate of encapsulated PP tissue influx (nmol/h) is described by Eqs. (14) and (15), which are in line with Eqs. (7) and (11), respectively:

14 $$ \mathrm{encapsulated}\ \mathrm{PP}\ \mathrm{liver}/\mathrm{spleen}/\mathrm{kidney}\ \mathrm{influx}={\mathrm{k}}_{\mathrm{PPB}\mathrm{X}}\times {\mathrm{V}}_{\mathrm{DPP}}\times {\mathrm{C}}_{\mathrm{PPB}}\left(\mathrm{t}\right) $$

15 $$ \mathrm{encapsulated}\ \mathrm{PP}\ \mathrm{tumor}\ \mathrm{influx}={\mathrm{k}}_{\mathrm{PPB}\mathrm{T}}\times {\mathrm{m}}_{\mathrm{T}}\times {\mathrm{C}}_{\mathrm{PPB}}\left(\mathrm{t}\right) $$

For comparison between the different tissues, the rate of influx was also expressed per gram of tissue by Eqs. (16) and (17):

16 $$ \mathrm{encapsulated}\ \mathrm{PP}\ \mathrm{influx}\ \mathrm{per}\ \mathrm{gram}\ \mathrm{of}\ \mathrm{liver}/\mathrm{spleen}/\mathrm{kidneys}=\frac{{\mathrm{k}}_{\mathrm{PPB}\mathrm{X}}\times {\mathrm{V}}_{\mathrm{DPP}}\times {\mathrm{C}}_{\mathrm{PPB}}\left(\mathrm{t}\right)}{{\mathrm{m}}_{\mathrm{X}}} $$

17 $$ \mathrm{encapsulated}\ \mathrm{PP}\ \mathrm{tumor}\ \mathrm{influx}\ \mathrm{per}\ \mathrm{gram}\ \mathrm{of}\ \mathrm{tissue}={\mathrm{k}}_{\mathrm{PPB}\mathrm{T}}\times {\mathrm{C}}_{\mathrm{PPB}}\left(\mathrm{t}\right) $$

Subsequently, the influx rates per tissue were calculated using Eq. (4) and (14) or (15), and the influx rates per gram of tissue were calculated using Eq. (4) and (16) or (17). The resulting rates were plotted versus time.

Differences between the rate of tissue influx or the rate of influx per gram of tissue for the various tissues were considered significant when there was no overlay between the [(k_PPBX_ ± SE) × (V_DPP_ or m_T_ ± SD)] intervals or $\frac{\left[\left({\mathrm{k}}_{\mathrm{PPBX}}\pm \mathrm{SE}\right)\times \left({\mathrm{V}}_{\mathrm{DPP}}\mathrm{or}\ {\mathrm{m}}_{\mathrm{T}}\pm \mathrm{SD}\right)\right]}{{\mathrm{m}}_{\mathrm{X}}\pm \mathrm{SD}}$ intervals, respectively, of two tissues. SE is the estimated standard error and SD is the estimated standard deviation.

#### Calculation of the rate of release

**Fig. 1:**
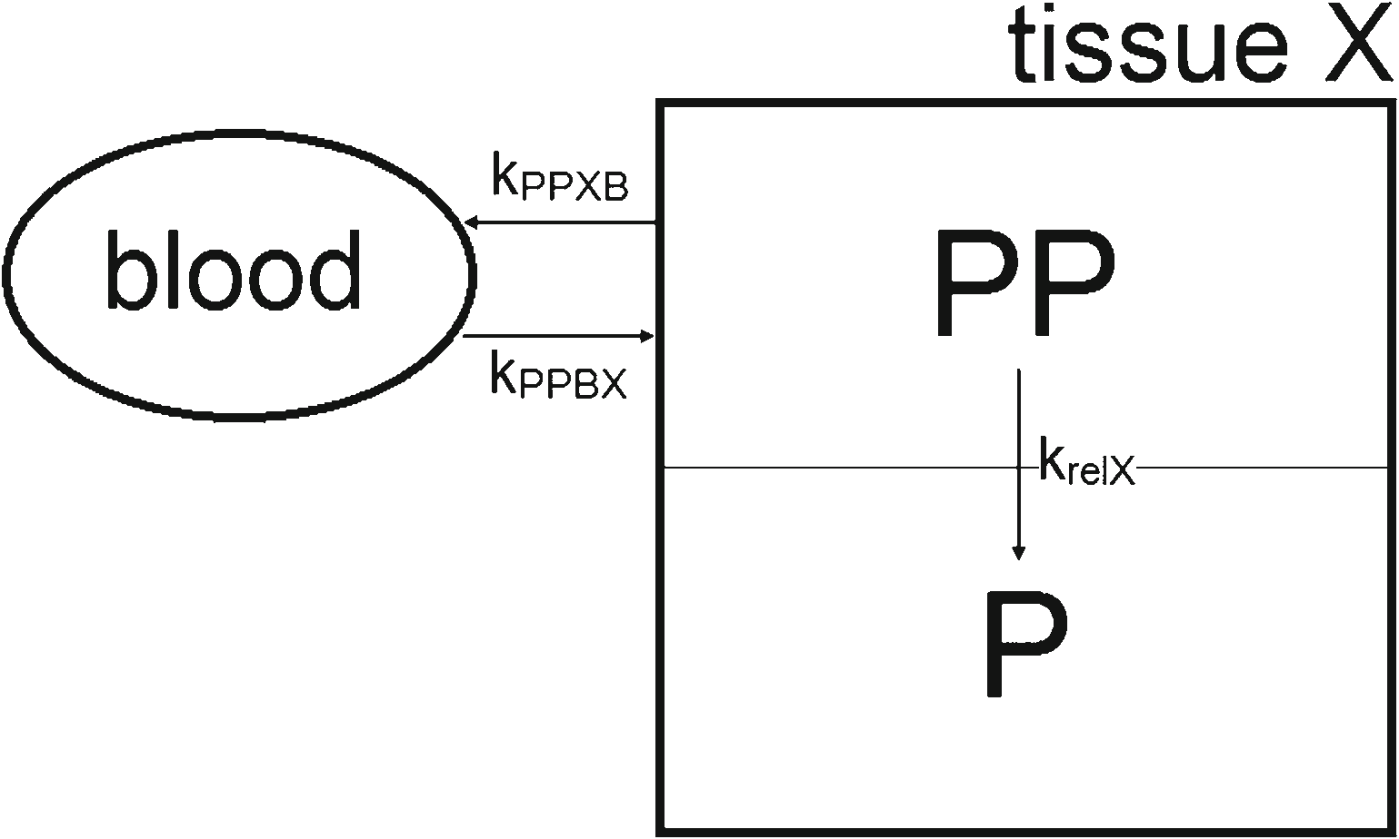
Basic representation of the kinetics of the drug when still encapsulated, where k_PPBX_ is the rate constant corresponding to the influx of encapsulated PP from the blood, PP is released from the liposomes according to the rate constant k_relX_, and k_PPXB_ represents the rate constant corresponding to the transfer of encapsulated PP to the blood. The transfer of encapsulated PP towards the blood is assumed to be negligible for all tissues studied (see section Calculation of the rate of release). The subsequent kinetics of the released drug is not included in the figure

Similarly, the rate of release of drug from the liposomes in the tissues of interest was determined as follows. A schematic representation of the kinetics of the drug when still encapsulated in a tissue “X” is shown in Fig. 1 and can roughly be divided into three processes: (1) influx of encapsulated PP from the blood into the tissue (k_PPBX_), (2) release of prednisolone phosphate from the liposomes (k_relX_), and (3) transfer of encapsulated PP from the tissue towards the blood (k_PPXB_). The accumulation of liposomes in tumor tissue is supposed to be unidirectional [3], because the lymphatic drainage in tumor tissue is highly reduced, which limits the clearance of the liposomes from the tumor and which improves liposomal tumor retention [4, 37]. Therefore, the transfer of encapsulated PP from the tumor to the blood (k_PPTB_) is assumed to be negligible. Furthermore, liposomes containing PP localize in phagocytes of the liver and spleen as observed by Schmidt et al. for a rat model of multiple sclerosis [38, 39]. And, while encapsulated PP is expected to be too large to be excreted by glomerular filtration [40], drug delivery systems with a diameter of about 75 ± 25 nm are believed to target the mesangial cells [40, 41]. Consequently, due to the digestic nature of these cells, it is likely that the fate of the majority of the liposomes ends in these phagocytes [29]. Therefore, in this study, the encapsulated PP is also expected to disappear from the liver, spleen and kidneys principally through drug release from the liposomes and the transfer of encapsulated PP from these tissues towards the blood is also assumed to be negligible. Thus, for the tissues of interest k_PPX_ is assumed to equal k_relX_.

The release of drug from liposomes in these tissues can be better described by a first-order process (see Results) and, therefore, the rate of release (nmol/h) derived from Eq. (7) or (11) is described by Eq. (18), which is in line with Eq. (7) or (11):

18 $$ \mathrm{rate}\ \mathrm{of}\ \mathrm{release}={\mathrm{k}}_{\mathrm{relX}}\times {\mathrm{m}}_{\mathrm{X}}\times {\mathrm{C}}_{\mathrm{PPX}}\left(\mathrm{t}\right) $$

For comparison between the different tissues, the rate of release was also expressed per gram of tissue by the following equation:

19 $$ \mathrm{rate}\ \mathrm{of}\ \mathrm{release}\ \mathrm{per}\ \mathrm{gram}\ \mathrm{of}\ \mathrm{tissue}={\mathrm{k}}_{\mathrm{relX}}\times {\mathrm{C}}_{\mathrm{PPX}}\left(\mathrm{t}\right) $$

Hereafter, the rates of release per tissue were calculated using Eq. (9) or (13) and (18), whereas the rates of release per gram of tissue were calculated using Eq. (9) or (13) and (19). The resulting rates were plotted versus time.

## Results

### Liposome characteristics

The liposome preparation used for i.v. administration contained 7.18 ± 0.06 mmol PP/L, of which 4.4% ± 0.3 was present as non-encapsulated drug. The presence of a minor non-encapsulated drug amount in liposome preparations is common [13, 14, 42]. After self-assembly of the liposomes, the non-encapsulated drug was removed by dialysis leaving a small amount of non-encapsulated drug in the preparation. The liposome diameter determined by DLS was 97 ± 30 nm. The dispersity was 0.077 indicating that the liposome preparation is monodisperse. The phospholipid content was 60 mM [22]. Due to the PEGylation and the small size these liposomes are optimized for delaying uptake by the organs of the MPS [22].

### Encapsulated PP and released P in the circulation

**Fig. 2:**
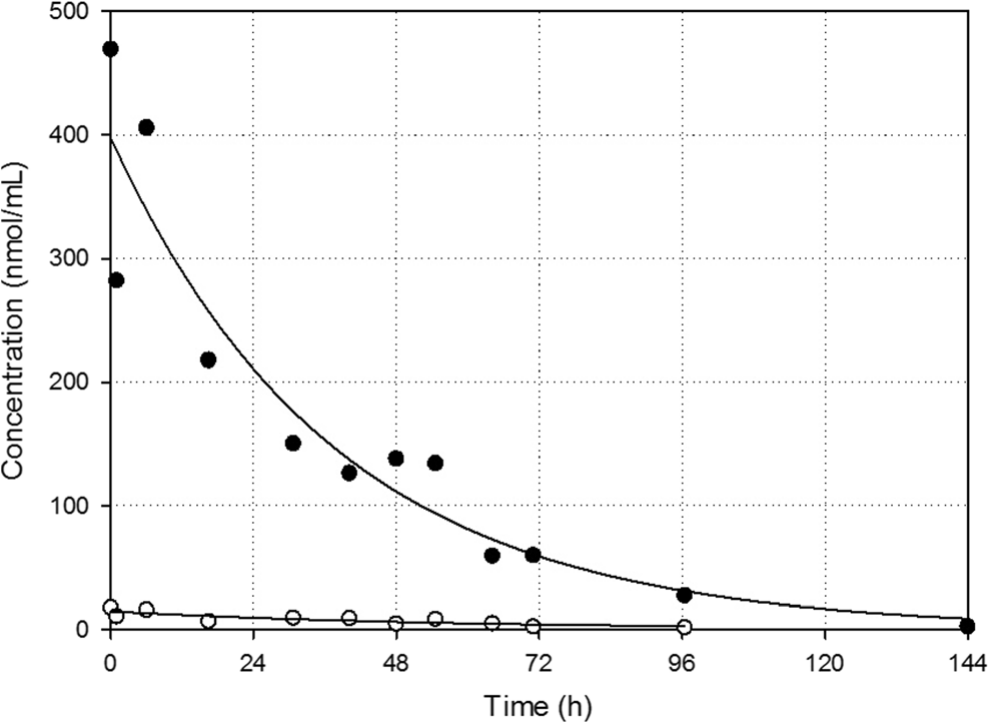
Individual blood concentrations of encapsulated PP (closed circles) and released P (open circles) with time after i.v. administration of 36 μmol/kg liposomal PP. The corresponding curve fits resulting from non-linear regression using Eqs. (4) and (5) are also plotted. The corresponding kinetic parameters are summarized in Table 1

The encapsulated PP and released P blood concentrations after i.v. administration of liposomal PP are shown in Fig. 2. The decline of the encapsulated PP as well as the released P concentration with time show first-order kinetics (the residuals are distributed normally: *p* value > 0.05). The corresponding kinetic parameters are summarized in Table 1. The calculated half-life of encapsulated PP and released P in the circulation are 26 h and 39 h, respectively. Similar results were observed previously in the plasma of rats with adjuvant arthritis after i.v. administration of 5 mg/kg PEGylated liposomal PP (≈10 μmol/kg) [22].

**Table 1:** Kinetic parameters and the corresponding estimated standard error (SE) or estimated standard deviation (SD) for liposomal PP in male C57Bl/6J mice bearing B16F10 melanoma tumors

		SE estimate	SD estimate
C_PPB_(0)	4.0 × 10^2^ nmol/mL	3 × 10^1^ nmol/mL	
k_PPB_	0.027 h^−1^	0.004 h^−1^	
C_PB_(0)^a^	15 nmol/mL	2 nmol/mL	
k_PB_	0.018 h^−1^	0.004 h^−1^	
V_DPP_^b^	2.2 mL		
k_PPBT_	0.004 mL/g/h	0.001 h^−1^	
k_relT_	0.03 h^−1^	0.01 h^−1^	
m_T_^c^	0.9 g		0.4 g
k_PPBL_	0.005 h^−1^	0.002 h^−1^	
k_relL_	0.3 h^−1^	0.1 h^−1^	
m_L_^d^	= a x t^2^ + b x t + c	g	
a	−0.00047	0.00005	
b	0.030	0.004	
c	1.22	0.05	
k_PPBS_	0.0033 h^−1^	0.0009 h^−1^	
k_relS_	0.7 h^−1^	0.1 h^−1^	
m_S_	0.06 g		0.02 g
k_PPBK_	0.0007 h^−1^	0.0001 h^−1^	
k_relK_	0.10 h^−1^	0.02 h^−1^	
m_K_	0.25 g		0.02 g

^a^$\frac{{\mathrm{C}}_{\mathrm{PB}}(0)}{{\mathrm{C}}_{\mathrm{PPB}}(0)+{\mathrm{C}}_{\mathrm{PB}}(0)}\times 100\%=3.5\%,$which is similar to the 4.4% non-encapsulated drug in the liposome preparation considering the SE

^b^Calculated from Fig. 2 and the dose

^c^No significant trend was observed for the mass of the tumor tissue with time. Quadratic regression of the tumor mass is not significant (*p* value 0.15)

^d^The mass of the liver varies with time. During the time frame for which the encapsulated PP concentrations are above the lower limit of quantification, the data is significantly not constant and is adequately described by a parabola (*p* values 0.000)

### Encapsulated PP and released P tissue concentrations

**Fig. 3:**
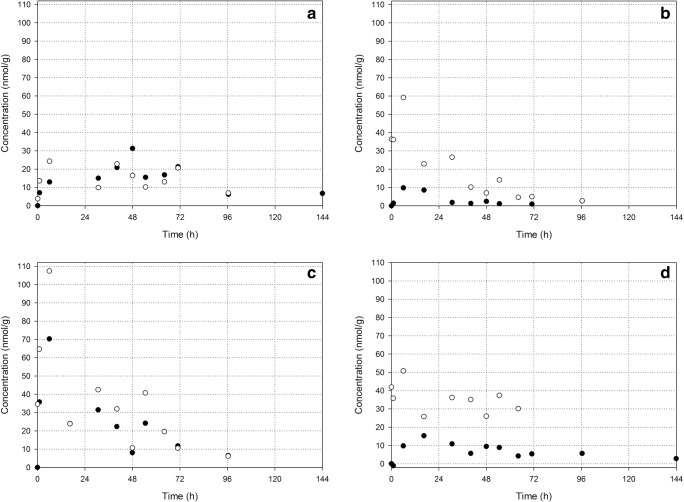
Individual concentrations of encapsulated PP (closed circles) and released P (open circles) with time in tumor (**a**), liver (**b**), spleen (**c**), and kidneys (**d**) after i.v. administration of 36 μmol/kg liposomal PP

The measured encapsulated PP and released P tissue concentrations were corrected for the drug located in the residual blood of the dissected tissues as described above. The resulting encapsulated PP and released P tissue concentrations are shown in Fig. 3.

**Fig. 4:**
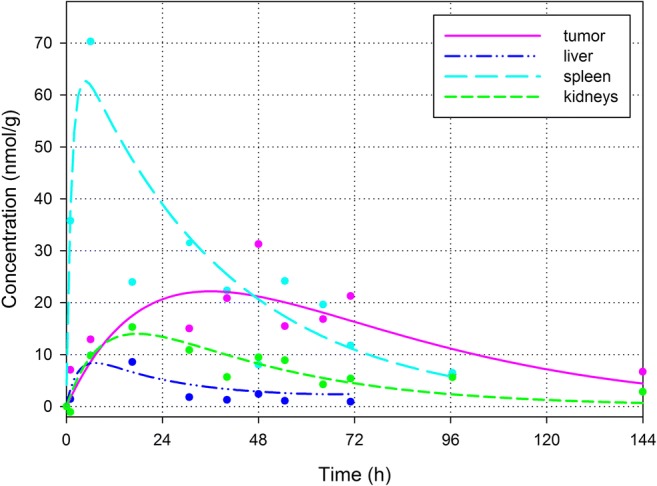
Non-linear regression of the encapsulated PP concentrations in tumor (purple; solid line), liver (dark blue; dash-dot-dot), spleen (cyan; long dash) and kidneys (green; short dash) according to Eqs. (9) and (13) describing a first order drug release. The corresponding values for the kinetic parameters are summarized in Table 1

Curve fits of the encapsulated PP tissue concentrations according to Eq. (9) and (13), which contain a first order rate of drug release, are shown in Fig. 4. The corresponding values for the kinetic parameters are summarized in Table 1. For all tissues, the encapsulated PP concentration is better described using a first order drug release (S-values 0.8–5.5) than by zero order release (S-values 1.9–13.3). The corresponding residuals are distributed normally (*p* value > 0.05). The largest encapsulated PP concentrations are observed in the spleen, for which the peak concentration is significantly larger than for tumor, liver and kidneys. The smallest encapsulated PP concentrations are observed in the liver, while the encapsulated PP tumor and kidney concentrations are in-between. These results are in line with the tissue distribution of ^111^In-labeled liposomes at 6 and 24 h after intravenous administration in B16F10 tumor-bearing C57Bl/6 mice [9].

**Fig. 5:**
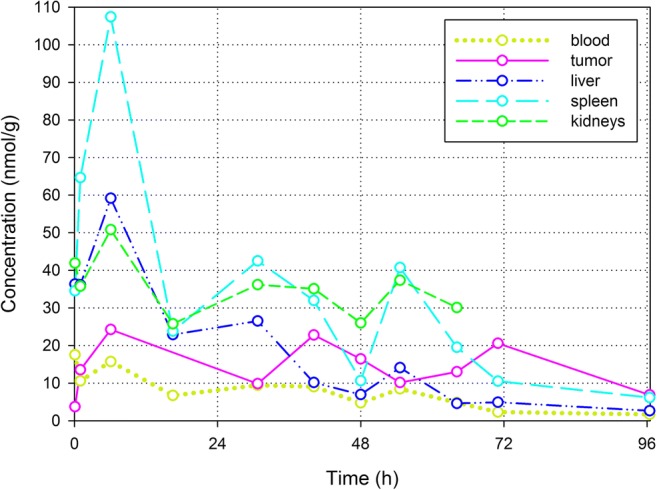
Released P concentrations in whole blood (lime; dotted line), tumor (purple; solid line), liver (dark blue; dash-dot-dot), spleen (cyan; long dash) and kidneys (green; short dash)

The released P blood and tissue concentrations are compared in Fig. 5. Released P is observed in whole blood as well as in all tissues of interest. The released P peak concentration at *t* = 6 h is significantly larger in the spleen than in the blood and the other tissues. Although it was not compared to tumor tissue, similar results were observed for hydrolyzed drug in liver, spleen and kidneys after administration of liposomal 4-methylumbelliferyl phosphate in mice [24]. The concentration in the tumor at *t* = 6 h is significantly smaller than the peak concentrations in the liver, spleen and kidneys. A significant difference between the concentrations in the tumor and blood at *t* = 6 h was not observed. However, the released P concentration is decreasing slower in the tumor than in the liver and spleen. At *t* = 96 h, the released P tumor concentration is no longer smaller than the released P concentration in the spleen and it is significantly larger than in the blood and liver.

### Tissue influx of encapsulated PP

**Fig. 6:**
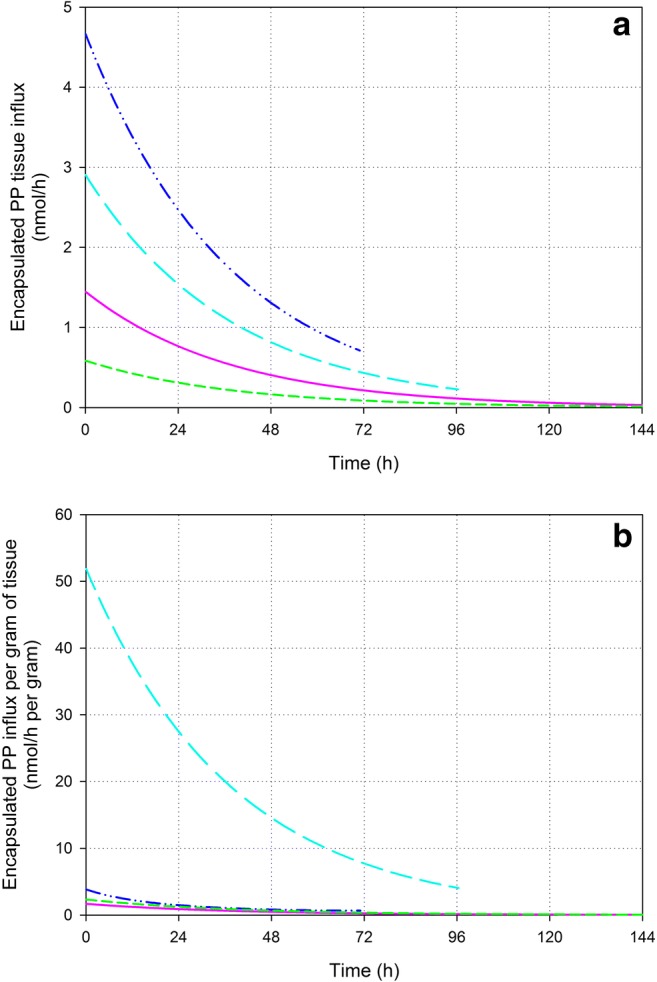
Calculated rate of tissue influx (**a**) and calculated rate of influx per gram of tissue (**b**) for encapsulated PP in tumor (purple; solid line), liver (dark blue; dash-dot-dot), spleen (cyan; long dash) and kidneys (green; short dash) by kinetic modelling

Non-linear regression of the encapsulated PP tissue concentrations showed that the tissue influx of encapsulated PP can be well described by a first order kinetic process for all tissues. The resulting kinetic parameters (see Table 1) were then used to model the rate of encapsulated PP tissue influx for each tissue, which is shown in Fig. 6. Although there seems to be a difference in rate of encapsulated PP tissue influx (see Fig. 6a), not all of them are significant probably due to the relatively large SD of the tumor mass (see Table 1). Large SD of the pre- and posttreatment tumor size were observed previously [9, 43]. No significant differences were observed for the influx towards the tumor as compared to the influx towards the liver, spleen and kidneys. Significant differences were observed for the smaller influx towards the kidneys as compared to the liver and spleen.

However, when observing the influx rate per gram of tissue a high preference of encapsulated PP for the splenic tissue becomes clear (see Fig. 6b). While no significant differences were observed between the encapsulated PP influx per gram of tumor, liver and kidneys, the significantly larger influx per gram of spleen is obvious.

### Drug release from the liposomes

**Fig. 7:**
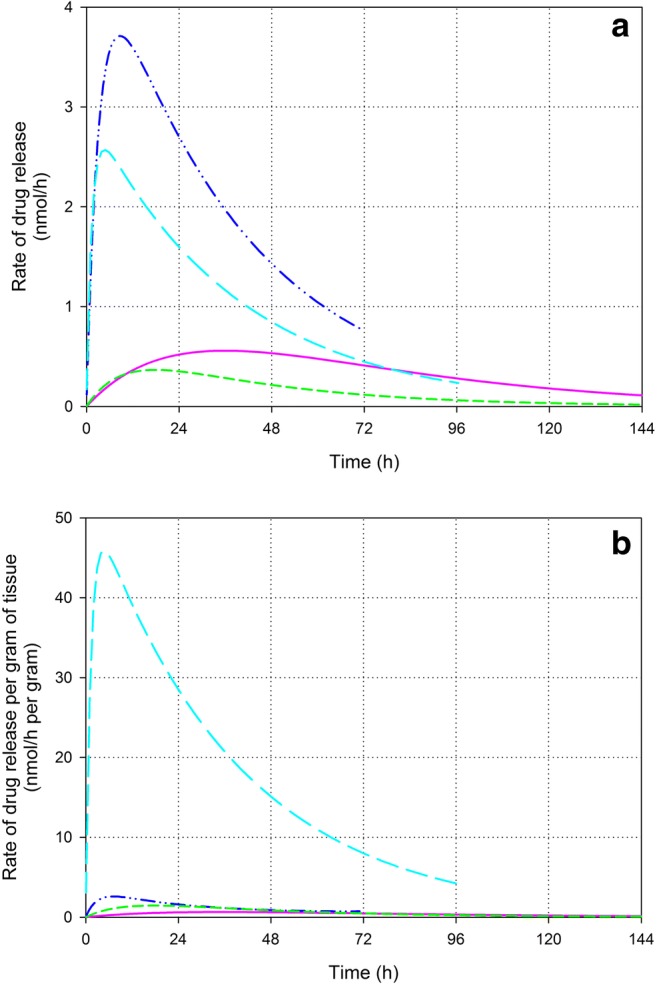
Calculated rate of drug release from liposomes per tissue (**a**) and calculated rate of drug release from liposomes per gram of tissue (**b**) in tumor (purple; solid line), liver (dark blue; dash-dot-dot), spleen (cyan; long dash) and kidneys (green; short dash) by kinetic modelling. Note, the rates of release are not normalized by the quantities of encapsulated PP present

As described above, the release of drug out of the liposomes can be better described by a first order kinetic process for all tissues. The corresponding kinetic parameters were used to model the rate of drug release from the liposomes as shown in Fig. 7. The rate of drug release from the liposomes in the tumor seems smaller but more extended as compared to the liver and spleen (see Fig. 7a). The extremely large capacity of the splenic tissue to release the drug becomes clear from the rate of drug release per gram of tissue shown in Fig. 7b: the maximal calculated rate of release per gram of spleen is about 74 x the maximal rate in tumor, about 17 x the maximal rate in liver and about 28 x the maximal rate in the kidneys.

## Discussion

### Extravasation of encapsulated PP towards the tumor versus the uptake by healthy tissues

Tissue concentrations of liposomes as well as the encapsulated drug depend on (A) the tissue influx and (B) the loss of the carrier or release of the encapsulated drug, respectively, in the tissue. Therefore, the tissue influx is considered a measure for the affinity of liposomes for a specific tissue. Here, the tissue influx of encapsulated PP is compared for the different tissues of interest. The encapsulated PP influx towards the tumor differs not significantly from the influx towards the liver, spleen and kidneys (as discussed above with regard to Fig. 6). Hence, the extravasation towards the tumor is in the same order of magnitude as the uptake by the other organs. An exception to this is the uptake per gram of spleen, which is significantly more pronounced. Since liposomal PP localizes in the macrophages of the liver and spleen [38, 39], probably, the uptake of liposomal PP by liver and spleen occurs mainly through uptake by tissue macrophages. Likely, although the liposomes are PEGylated, uptake by macrophages is still prominent. This is because PEGylation only slows down MPS uptake and does not avoid MPS uptake [4].

Figure 6b shows the high preference of encapsulated PP for the splenic tissue. Besides the larger macrophage density in the spleen as compared to most other tissues like the liver [44, 45], this is most probably due to the characteristics of the spleen. The spleen provides an enormous contact surface area and is able to reduce the blood flow yielding low shear rates of the liposomes, increased retention of the particles and prolonged contact with the macrophages [45].

### Drug targeting by liposomes

As discussed before, efficacy and toxicity are not determined by the liposomal concentration. Only released drug may induce efficacy/toxicity. Most probably, after uptake of liposomes by macrophages, PP is liberated in the endosomal/lysosomal compartment where it is dephosphorylated into P [29, 30]. Because of the previous observed efficacy [9, 29] and because P can easily pass membranes [46], it is assumed that the released P is not trapped in the lysosome, but can be available intra- and possibly also extracellularly [31]. Hence, the released P tissue concentrations shown in Figs. 3 and 5 are a measure of drug targeting. Note that, from a quantitative point of view, tumor targeting by released P is not more pronounced than the targeting of the liver, spleen and kidneys. The released P peak concentration at *t* = 6 h is even significantly smaller in the tumor than in the liver, spleen and kidneys. However, the released P concentration in the tumor tissue is more persistent compared to liver and spleen.

The released P tissue concentrations are a result of the liposomal tissue influx, the subsequent drug release out of the liposomes and the subsequent fate of the released P (i.e. retention, distribution, metabolism, excretion). The rate of drug release from the liposomes in the tumor seems smaller but more extended than in the liver and spleen (see Fig. 7), which is in accordance with the more persistent released P tumor concentrations observed. Possibly, this is due to (1) a smaller amount of macrophages in the tumor as compared to the liver and spleen, (2) different types in the tumor macrophage population as compared to the liver and spleen, and/or (3) the difficult accessibility of macrophages in distant areas of the tumor tissue. An extremely large rate of release is calculated per gram of spleen (see Fig. 7b). However, the released P spleen concentrations remain relatively small considering this extremely large release rate. Either P is rapidly metabolized in the spleen, and/or, more probably, the released P distributes quickly out of the spleen, which is to expect from a compound like P exhibiting biopharmaceutical properties to pass membranes [46].

In fact, a rapid redistribution of released P is expected in general and it would explain the observed P concentrations in the blood as shown in Fig. 2 as hypothesized previously by Schiffelers et al. [9, 22]. As discussed above, about 4% of the drug in the liposome preparation was not encapsulated. Since the half-life of P in mice is short, i.e. 16 min after i.v. administration of 10 μmol/kg in female Balb/c mice (internal study), P originating from the non-encapsulated drug amount in the liposome preparation leaves the circulation quickly within a couple of hours. In contrast, the netto P blood concentration after liposome PP administration is decreasing more gradually yielding a pseudo half-life of 39 h. Such P concentrations were also observed in the plasma (max 0.9% of the sum total concentration) after i.v. administration of 5 mg/kg liposomal PP (≈10 μmol/kg) in rats with adjuvant arthritis [22] and can be explained as follows. Since liposomal PP is probably stable in the circulation [22], it suggests that P is introduced to the circulation by redistribution of released P from spleen and other tissues for multiple days. This is supported by the relatively large and persistent P concentrations in the kidneys in comparison to the calculated drug release per gram of kidneys. Basically, it reveals a mechanism of sustained release for liposomal PP.

P itself exhibits nonlinear pharmacokinetics in plasma mainly due to its nonlinear protein binding but also to reversible metabolism between P and prednisone [26, 47, 48]. P is predominantly metabolized to the inactive metabolite and pro-drug prednisone in the liver [48–50] and to some extend in other organs such a kidney [51]. In addition, other phase 1 metabolites of P are formed, e.g. 20ß-hydroxyprednisone, 6ß-, 20α- and 20ß-hydroxyprednisolone [52, 53], which are conjugated or not conjugated [54], and rapidly excreted via the kidney together with a considerable amount of unchanged prednisolone. Metabolism of P (after PP release from the liposomes and conversion to P) was out of scope of this study.

## Conclusions

To our knowledge, accurately measured released drug concentrations in solid tumors and in healthy tissues have not been compared after administration of liposome formulations. Combined with kinetic analysis, it provides important insights into the pharmacokinetics of liposomal drug delivery systems.

The calculated rate of tumor influx of encapsulated PP appears not to be significantly larger compared to that for the other tissues. Subsequently, the rate of drug release from the liposomes seems not larger in the tumor than in the liver and spleen and the released P peak concentration at *t* = 6 h is smaller in the tumor than in the other tissues. From a quantitative point of view, tumor targeting by released P is not more pronounced. However, drug release in the tumor seems more extended and the netto released P concentration decreases more slowly in the tumor than in the liver and spleen.

A high capacity with regard to the encapsulated PP tissue influx as well as the drug release from the liposomes is calculated for the spleen. This is probably due to the anatomy and high macrophage density of the spleen. Likely, the released P in the spleen (and possibly also in other tissues) is quickly redistributed towards the blood and other tissues.

A fast redistribution of the released drug counteracts the targeted drug delivery by the liposomes. However, drug release in the tumor seems to be maintained for an extended period and at *t* = 96 h the released drug concentration in the tumor is significantly larger than that in the central circulation. This in contrast to the released P concentration at *t* = 96 h in the liver and spleen. These extended release characteristics in the tumor probably contribute to the beneficial effect. It should be noted however that higher released drug concentrations are formed in other tissues.

## Electronic supplementary material

ESM 1 (PDF 412 kb)
