# Supplementary material for: The availability of drug by liposomal drug delivery: Individual kinetics and tissue distribution of encapsulated and released drug in mice after administration of PEGylated liposomal prednisolone phosphate
Source: Invest New Drugs. 2018 Dec 13;37(5):890–901. doi: 10.1007/s10637-018-0708-4 (PMC6736927; doi:10.1007/s10637-018-0708-4)
Supplement: Supplementary file 1 — (PDF 412 kb) [file 10637_2018_708_MOESM1_ESM.pdf]

Invest New Drugs

DOI 10.1007/s10637-018-0708-4

The availability of drug by liposomal drug delivery

Individual kinetics and tissue distribution of encapsulated and released drug in mice after administration of PEGylated liposomal prednisolone phosphate

Evelien A.W. Smits[a*], José A. Soetekouw[b], Ebel H.E. Pieters[a], Coen J.P. Smits[c], Nicolette de Wijs-Rot[d], Herman Vromans[e]

[a]Department of Pharmaceutical Sciences, Utrecht University, Utrecht, The Netherlands

[b]Quality Control, Nutricia Early Life Nutrition, Cuijk, The Netherlands

[c]Holland Innovative, Eindhoven, The Netherlands

[d]Education and Student Affairs, Eindhoven University of Technology, Eindhoven, The Netherlands

[e]Department of Clinical Pharmacy, Division of Laboratory Medicine & Pharmacy, University Medical Centre Utrecht, Utrecht, The Netherlands

*Address for correspondence: P.O. Box 80082, 3508 TB Utrecht, The Netherlands.

E-mail address: e.a.w.smits@gmail.com

Supplementary material - ESM 1

Tumor pharmacokinetics of encapsulated drug

Non-linear regression of the tumor concentration of encapsulated prednisolone phosphate (encapsulated PP) versus time was performed using equation (8) and (9) as described in the article. Note, equation (8) and (9) were derived from equations (6) and (7), which express the change of the amount of encapsulated PP with time. Fig. S1a shows there is no trend discerned between the amounts of encapsulated PP in the tumor and the time, whereas a relationship between the encapsulated PP concentration and the time is evident from Fig. S1b. Moreover, non-linear regression of the encapsulated PP tumor concentrations using equations (12) and (13) results in better fits. These equations were derived from differential equations (10) and (11) describing the change of the encapsulated PP tumor concentration with time. The corresponding S-values were 6.3 (zero order release) and 5.5 (first order release) for the concentration-based regression models, compared to 9.5 and 9.3, respectively, for the amounts-based regression models.

Differences between the results obtained for amounts- and concentration-based regression models can occur when the mass of the tissue is not constant with time as is expected for the tumor tissue. In this case, k_{PPBT} and k_{relT} are rather constant per gram of tumor and not per tumor as such. This indicates that the amounts of entities causing the different kinetic processes is rather similar per gram of tumor and not per whole tumor. With regard to the encapsulated PP tumor influx, this is in line with tumor physiology: during tumor growth new blood vessels with wide fenestrations, through which liposomes extravasate [S1], are formed [S2]. Thus, upon tumor growth the amount of entrances for liposomes also increases.

When comparing equation (15) describing the rate of tumor influx and equation (17) describing the rate of influx per gram of tumor to the corresponding equations for the other tissues ((14) and (16), respectively), such kinetics for the influx of encapsulated PP towards the tumor appears to be a handicap. Assuming whole blood and tissue densities are 1 g/mL, V_{DPP} is about 2.5 times m_T and $\frac{V_{DPP}}{m_{L/S/K}}$ is always larger than one. Consequently, the encapsulated PP influx towards the tumor and the encapsulated PP influx towards one gram of tumor, respectively, are at a quantitative disadvantage.

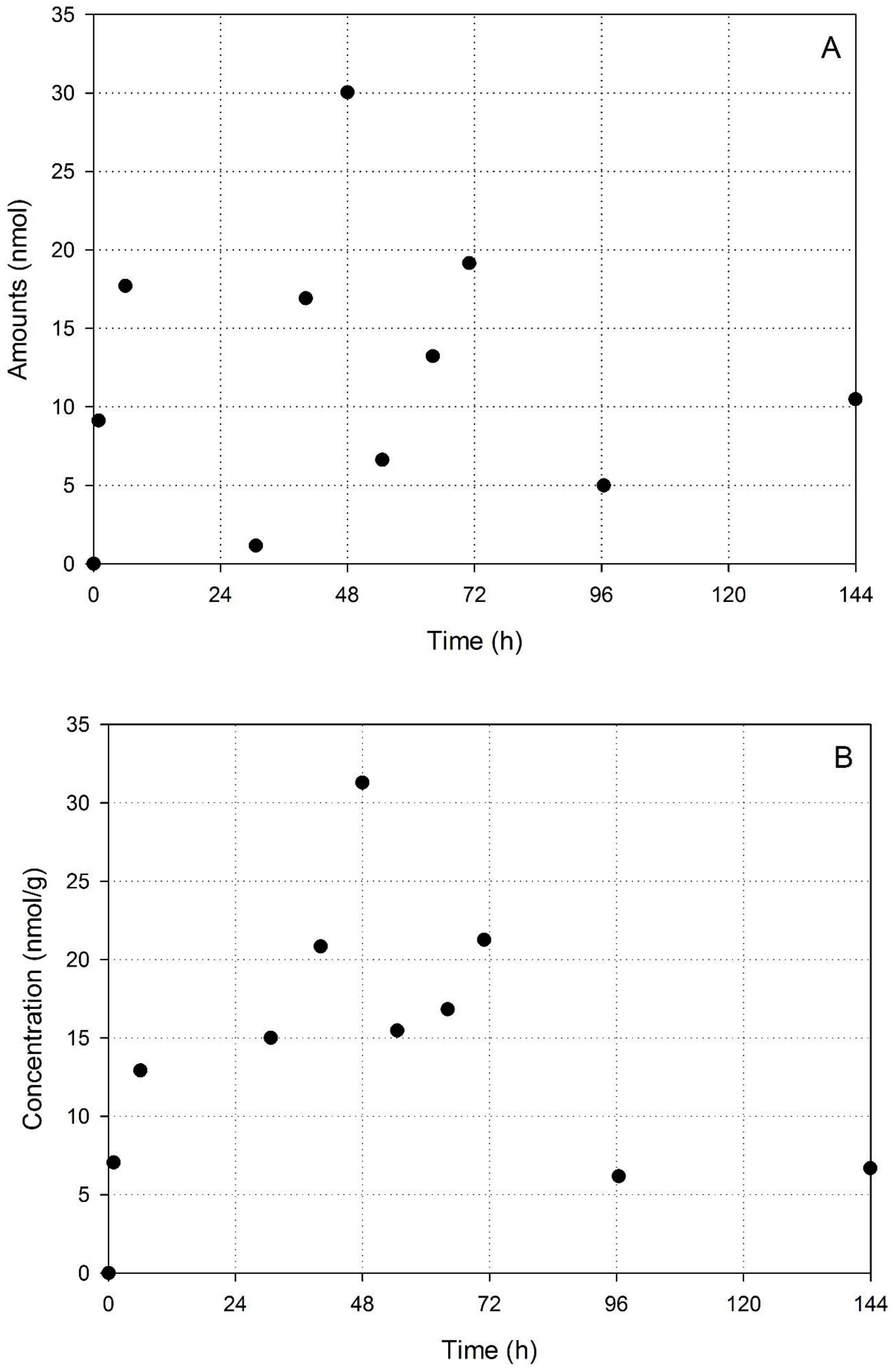

Fig. S1 Amounts (a) and concentrations (b) of encapsulated PP in the tumor as function of the time.

Hepatomegaly

The mass of the liver was not constant with time (see article, Table 1). Most plausible, the observed increase in liver mass is caused by hepatomegaly due to hepatocytic accumulation of glycogen, which was also observed after short term high dose corticosteroid therapy [S3], and was caused by prednisolone released by macrophages in the liver.

References

S1 Nichols JW, Bae YH (2014) EPR: evidence and fallacy. Journal of Controlled Release 190:451–464

S2 Nishida N, Yano H, Nishida T, Kamura T, Kojiro M (2006) Angiogenesis in cancer. Vascular Health and Risk Management 2:213–219

S3 Iancu TC, Shiloh H, Dembo L (1986) Hepatomegaly following short-term high-dose steroid therapy. Journal of Pediatric Gastroenterology and Nutrition 5:41-46
